# A systematic review and meta-analysis of shRNA–IL-6-engineered CAR-T cells for B-cell acute lymphoblastic leukemia: a stepping stone toward risk-free immunotherapy

**DOI:** 10.1042/BSR20260143

**Published:** 2026-05-22

**Authors:** Mohamed S. Attia, Brett Dyer, Nigel McMillan, Matthew Zunk

**Affiliations:** 1 School of Pharmacy and Medical Science, Griffith University, Southport 4215, Queensland, Australia; 2 Griffith Biostatistics Unit, Griffith Health, Griffith University, Southport 4215, Queensland, Australia; 3 Institute for Biomedicine and Glycomics, Griffith University, Southport 4215, Queensland, Australia

**Keywords:** CAR T therapy, CD19, Cytokine release syndrome, immune-related adverse effects

## Abstract

Clinical application of chimeric antigen receptor (CAR)-T cells, especially those targeting CD19, stands as a breakthrough in treating relapsed or refractory B-cell acute lymphoblastic leukemia. Yet, preventing immune-related adverse events, like severe cytokine release syndrome (CRS) and immune effector cell-associated neurotoxicity syndrome (ICANS), remains a significant concern. This meta-analysis looked at the efficacy and safety of interleukin-6 knockdown CAR-T therapy. The study’s primary outcomes included the incidence of CRS, ICANS, and the number of patients achieving an early complete response (CR) and overall response (OR) rates at one-month post-infusion of anti-CD19 shRNA-engineered CAR-T cells. The random-effects model was used to estimate summary effects. Certainty of evidence was assessed using GRADE. Out of 275 studies screened, 7 studies were eligible (*n* = 178 patients). The pooled OR and CR rates were 88% (95% CI: 81–92) and 84% (95% CI: 78–89), respectively, with no heterogeneity detected. Among 147 patients, 116 (78%, 95% CI: 68–85) developed CRS, whereas 46 (28%, CI: 21%–35%) out of 178 were affected by severe grades (≥3). While ICANS was detected in 13 out of 159 patients (13%, CI: 2%–51%, *I*^2^ = 69.5%), three studies confirmed the absence of severe grade ICANS. According to GRADE assessment, current analysis presents low certainty of evidence supporting investigated outcomes, except for ICANS (any grade) that was deemed very low. More importantly, as all included studies were conducted in China, the findings may not be readily generalizable to other healthcare systems and ethnically diverse populations. Therefore, our confidence in the effect estimates is limited and it may vary from true estimates.

## Introduction

Hematologic malignancies have been transformed with the advent of chimeric antigen receptor T-cell (CAR-T) immunotherapy, yielding unprecedented outcomes in patients with otherwise relapsed/refractory status [1,2]. This sophisticated cellular engineering approach involves the *ex vivo* modification of autologous T lymphocytes to express a synthetic CAR, enabling precise recognition and potent eradication of malignant cells [3,4]. Conventional CAR-T (cCAR-T) constructs possess profound therapeutic efficacy, yet their broader clinical adoption remains limited. Among the concerns related to CAR-Ts’ applicability is the possibility of serious immune-related adverse events (IrAEs), particularly cytokine release syndrome (CRS), a potentially fatal complication of CAR-Ts therapy resulting from an acute surge in pro-inflammatory cytokines [5]. Immune effector cell-associated neurotoxicity syndrome (ICANS) is another IrAE of CAR-Ts therapy, which is characterized by a range of symptoms from mild confusion to critical cerebral edema, necessitating specialized neuromonitoring. These systemic inflammatory and neuroinflammatory sequelae, driven by the robust induction of CAR-Ts’ proliferation and subsequent cytokine cascades, underscore a critical need for refined engineering strategies that mitigate off-target toxicities.

cCAR-T manufacturing paradigms predominantly leverage integrating viral vectors, such as lentiviral vectors, to achieve stable and constitutive expression of the CAR transgene within the T-cell genome [6]. While this genomic integration confers durable CAR expression and sustained anti-tumor persistence, it concomitantly predisposes patients to protracted and potentially life-threatening CRS and ICANS due to the unremitting activity of the engineered T cells and the persistent secretion of pro-inflammatory cytokines [7]. The irreversible nature of lentiviral gene integration further complicates the management of severe toxicities, as the engineered cellular product cannot be readily controlled or eliminated post-infusion. This consequently calls for pharmacologic interventions that may inadvertently compromise anti-tumor immunotherapeutic efficacy.

Despite being common IrAEs of CAR-T therapy, CRS and ICANS have distinct mechanisms, although they often occur together [5]. CRS is initiated when CAR-T cells recognize tumor antigens and release proinflammatory cytokines such as IL-2, interleukin-6 (IL-6), and TNF-α [8,9]. Further, this surge in cytokine levels precipitates a cascade of immune reactions, activating endothelial cells, leading to increased vascular permeability, fluid leakage, and organ dysfunction [10,11]. This process is further exacerbated by tumor cell pyroptosis [12], which releases damage-associated molecular patterns (DAMPs) [13], activating macrophages, and in turn releasing additional inflammatory cytokines [9]. This systemic inflammation can eventually culminate in coagulopathy, organ failure, and death in severe grades of CRS [14,15]. Typically, ICANS occurs following CRS and is characterized by a disruption of the blood-brain barrier, marked with affected cerebral endothelial cells, allowing immune cells and cytokines to infiltrate the brain [16,17]. Cytokine infiltration through the BBB contributes to neuroinflammation, neurological dysfunction with symptoms of cognitive disturbances, seizures, and cerebral edema [18]. Inflammatory cascades in the brain are further exacerbated by activated microglia and other myeloid cells, yielding severe neurotoxicity [19–21]. Tackling both of these IrAEs often necessitates integrated supportive care along with corticosteroids and targeted therapies to control cytokine release [22].

In light of these inherent safety challenges, innovative CAR-T engineering approaches have been developed to enable more precise control over CAR expression kinetics and cellular activity. Short hairpin RNA (shRNA)-engineered CAR-Ts or ssCAR-Ts provide a sophisticated genetic mechanism for targeted immunomodulation. As evidenced by recent clinical investigations, shRNA constructs can be precisely designed to selectively down-regulate the expression of specific genes implicated in the pathogenesis of CRS and ICANS, such as those encoding key pro-inflammatory cytokines like IL-6 or interferon-γ (IFN-γ) [23,24]. As discussed earlier, IL-6 has a central role in both CRS pathophysiology and neurological disruption underlying ICANS, which explains the mechanistic rationale for targeting IL-6 knockdown via shRNA-designed anti-CD19 CAR-Ts (ssCAR-Ts) and justifies the focus of this meta-analysis on CRS and ICANS as the primary safety endpoints. Further, studies have demonstrated that shRNA-mediated silencing of IL-6 in anti-CD19 CAR-Ts (ssCAR-T-19) significantly reduces the frequency and severity of CRS and neurotoxicity in patients with relapsed or refractory B-cell acute lymphoblastic leukemia disease (r/r B-ALL) [25–27]. This targeted gene silencing strategy enables a direct mitigation of the cytokine storm at its source, thereby reducing the severity of systemic and neuroinflammatory toxicities while judiciously preserving the essential anti-tumor effector functions of the CAR-Ts. This integrated safety mechanism offers a superior therapeutic profile by decoupling anti-tumor efficacy from severe adverse events, representing a significant advancement over traditional lentiviral methods.

Given the promising preclinical and early-phase clinical data supporting the enhanced safety profiles of ssCAR-Ts, a comprehensive synthesis of the available evidence is essential to fully elucidate their benefits and future clinical implications. This systematic review and meta-analysis aims to assess how effective and safe the ssCAR-Ts are in r/r B-ALL, specifically focusing on their capacity to attenuate their IrAEs. Herein, this review integrate data from studies employing RNAi technologies to construct ssCAR-Ts. By doing so, we seek to inform future clinical development of optimized therapeutic use of advanced CAR-T platforms, in turn facilitating the recognition of safer, reliable, and more feasible cellular immunotherapies.

## Methods

### Systematic search and selection criteria

To identify clinical studies evaluating ssCAR-Ts in hematologic malignancies, we conducted a systematic search in four major databases: PubMed, Embase, Scopus, and Web of Science from inception to 1 July 2025, with no restrictions on language or publication date. Search strategies were designed to incorporate controlled vocabulary terms (i.e., MeSH in PubMed, Emtree in Embase), as well as free-text keywords pertaining to shRNA, RNA interference, gene silencing, CAR-T cell therapy, and hematologic malignancies. Boolean logic was applied to titles, abstracts, keywords, and indexing fields where appropriate to optimize comprehensiveness and precision. Furthermore, we manually reviewed the reference lists of included studies and relevant reviews to identify any potential records eligible for inclusion. The complete database-specific search strategies are listed in Supplementary Table S1. Our review of references yielded relevant single-center and multi-institutional studies reporting on patient outcomes after receiving IL-6 shRNA-modified CAR-Ts for refractory or relapsed B-ALL. Covidence facilitated the removal of duplicates, screening of studies, and data extraction from published articles or conference proceedings. Study selection and data extraction were performed by a single reviewer (M.S.A.), who screened titles and abstracts for eligibility based on pre-defined inclusion criteria and extracted relevant data. Data were collected on the study and participants’ characteristics, intervention dosing details, and outcome measures, along with funding sources. Missing or unclear information was assumed to be either not reported or not applicable.

### Eligibility criteria

In our study, the eligibility criteria were based on the PICO framework, wherein the population of interest included patients diagnosed with CD-19-positive B-ALL. The disease state was relapsed or refractory, defined according to the National Comprehensive Cancer Network (NCCN)® Clinical Practice Guidelines for ALL (NCCN; Version 2.2025) [28], where relapsed disease was the reappearance of leukemic blasts (>5% in bone marrow) or extramedullary disease, and refractory disease was failure to achieve complete remission following at least one prior line of therapy. Patients treated with IL-6 knockdown anti-CD19 CAR-T cells (ssCAR-T-19) were exclusively involved in the analysis. Studies were eligible even if they did not have a comparison group.

Among the primary efficacy endpoints was the overall response (OR) rate, which is described as the proportion of patients who reached a complete or partial remission. Meanwhile, the complete response (CR), or remission rate, was defined as less than 5% bone marrow blasts with no extramedullary disease; in patients with CNS involvement, remission further required clearance of leukemic blasts from the CSF and resolution of neurological symptoms. Also, minimal residual disease (MRD) negativity was defined as less than 0.01% bone marrow blasts by multiparameter flow cytometry. In addition, other endpoints that were insufficient for quantitative analysis such as progression-free survival (PFS) and duration of response (DOR) were discussed in the narrative synthesis. Meanwhile, the primary focus of the safety analysis was on CRS and ICANS, each stratified by low-grade and high-grade (≥3 events). For each study, the grading systems used to classify CRS and ICANS severity were extracted, including criteria from the American Society for Transplantation and Cellular Therapy (ASTCT 2019), the National Cancer Institute CTCAE, local institutional systems, or cases where the system was not specified. The total number of patients in each study, the number of events, and the corresponding incidence proportions were extracted for each outcome. Additionally, since most studies did not provide explicit numerical data for inflammatory cytokines, such as IL-6, IL-2, and TNF-α, their results were discussed narratively.

In such cases, when overlapping reports originated from the same study population, outcome-specific data were extracted from the most relevant publication for each endpoint to avoid duplication. The data were either directly extracted from studies reporting efficacy and safety results or manually derived from figure digitization using graph2table.com.

### Statistical analysis

The data synthesis was conducted based on event rates (events/total) for outcomes of interest from each study cohort. The event rates were pooled, using random-effects model. Before pooling, the Freeman–Tukey double arcsine transformation was performed to stabilize the variance of proportions. Forest plots were used to visualize estimates from individual studies and assess heterogeneity. Further, heterogeneity among studies was quantified using the *I*^2^ statistic, categorized as low (≤25%), moderate (25–50%), high (50–75%) or very high (>75%) heterogeneity. A 95% prediction interval was then calculated to provide an estimate of the range of possible outcome estimates that would be expected in future studies. Data analysis was conducted in R version 4.4.2 [29].

### Sensitivity analysis

Sensitivity analyses using leave-one-out procedures and Baujat plots were performed to explore sources of high heterogeneity and assess the influence of individual studies on pooled estimates.

### Quality assessment of included studies

The quality of each study was evaluated using the NIH quality assessment checklist for pre-post studies with no control group, which was selected for its suitability to the design of the included studies [30]. Twelve NIH quality criteria were used to evaluate pre-post studies, including a clear objective, clear eligibility criteria, representative participants, full enrollment of eligible participants, adequate sample size, consistent interventions, validity and consistency of outcomes, blinded assessors, ≤20% loss to follow-up, pre-post statistical comparison, pre- and post-measurements (time series), and individual data for group interventions. As part of the quality assessment process, M.S.A. and B.D. conducted independent evaluations of included studies, with final quality scores being determined after consultation with M.Z. and N.M. Studies were then rated as good, fair, or poor, in accordance with the previous literature [31].

### Certainty of evidence

For assessing the certainty of evidence supporting each outcome, the GRADE framework was used in accordance with the GRADE handbook [32,33]. Using this framework, M.S.A. and review team evaluated evidence based on five downgrading domains: study limitations, inconsistency, indirectness, imprecision, and publication bias, as well as three elements for upgrading (effect size, residual confounding, and exposure–response gradient). Due to the nature of the evidence being pooled and study design, upgrading domains were not applicable in assessment. Based on the study design, the initial ratings for each outcome were assigned as recommended by the GRADE handbook. Eventually, the level of overall certainty was determined by considering the NIH risk of bias scores, tallies of association direction, raw data, and forest plots, and it was assigned a final rating between very low, low, moderate, or high.

## Results and discussion

**Figure 1:**
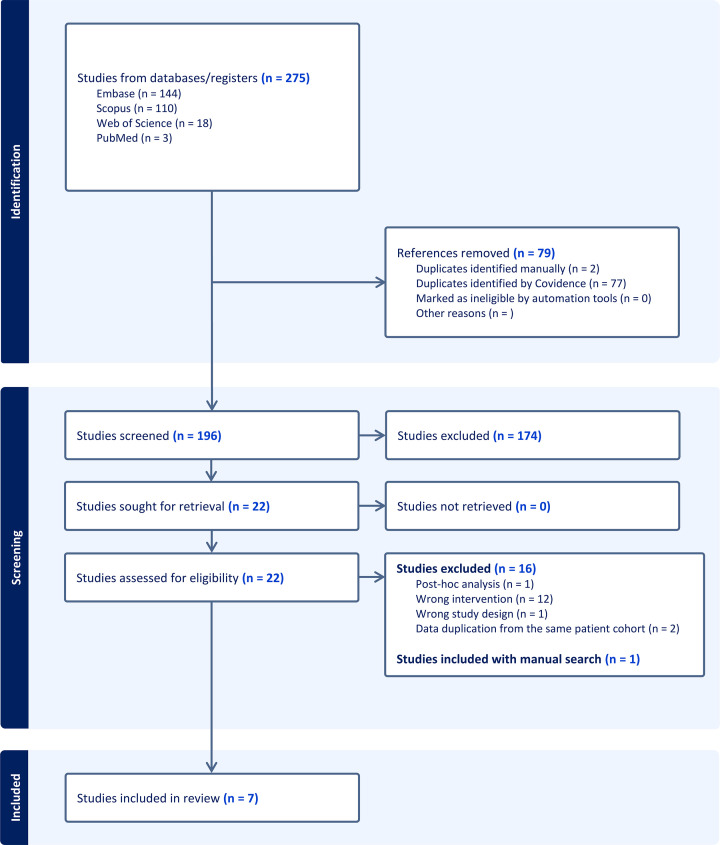
PRISMA flow diagram summarizing study identification, screening, and inclusion. Of 275 records retrieved across four databases, 22 underwent full-text review after duplicate removal and screening. Sixteen were excluded (wrong intervention, n = 12; duplicate cohort data, n = 2; post-hoc analysis, n = 1; wrong design, n = 1), and one additional study was identified via manual reference screening, yielding seven studies for qualitative and quantitative synthesis.

After conducting a systematic literature search, 275 potentially relevant studies were identified (Figure 1). Prior to screening, 79 duplicates were initially detected and removed using Covidence. After title and abstract screening, 22 full-text articles were screened, 16 of which did not satisfy eligibility criteria. Two studies were excluded as they reported data from the same patient cohort at an earlier cutoff date [34,35]. In total, seven studies were included, comprising 178 patients with relapsed or refractory B-ALL who received ssCAR-T therapy. Among the included studies, five were single-arm studies and one was a small pilot with a concurrent cCAR-T arm (Kang 2019), and the other one compared its ssCAR-T cohort against a separate contemporaneous cCAR-T cohort (Kang 2023). Only single-arm ssCAR-T data were extracted. Most included studies were peer-reviewed journal publications (71.4%); the remaining studies were two conference abstracts (28.6%). All the studies included in the present review used a single type of CAR-T construct incorporating co-stimulation with CD19, and five of them explicitly evaluated autologous CAR-T products.

**Figure 2:**
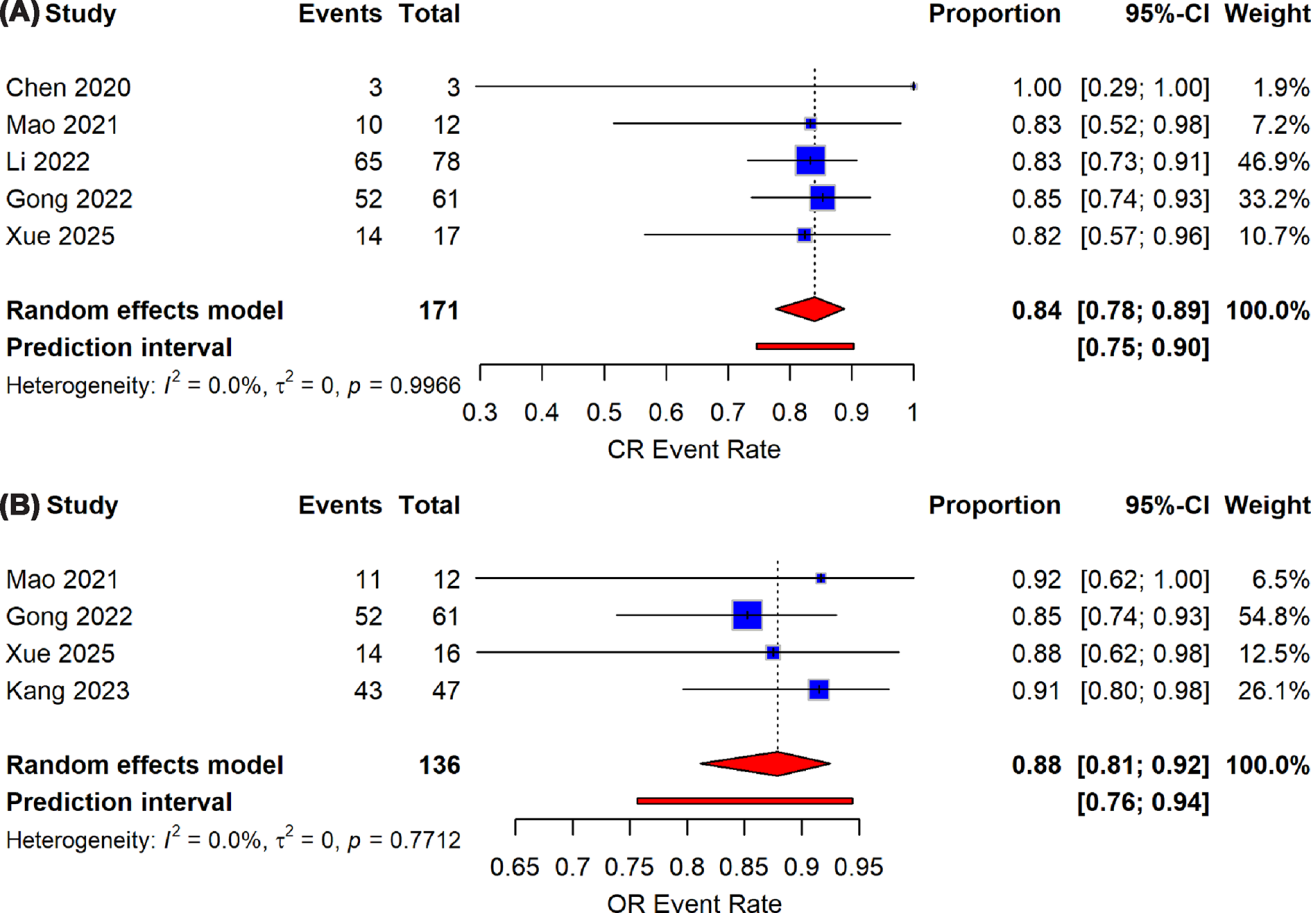
Pooled efficacy outcomes of ssCAR-T-19 therapy in r/r B-ALL at one month post-infusion. Forest plots of (**A**) complete response (CR) and (**B**) overall response (OR) rates across included studies.

The efficacy of ssCAR-T therapy was evaluated and reported across included studies, highlighting complete OR and CR rates as key outcomes (Table 1). ssCAR-Ts demonstrated a noticeably high early OR across multiple studies, with Gong et al. [26] reporting 85% complete CR, Mao and Xu [36] achieving 91.6%, Xue et al. [37] showing 88%, and Kang et al. [25] reaching 91.5% (Figure 2B). Overall, ssCAR-Ts achieved a high pooled OR of 88% (95% CI 81–92) with a 95% prediction interval ranging from 76% to 94%, suggesting a relatively narrow and consistent effect size across the analyzed studies.

**Table 1:** Summary of study characteristics for studies reporting efficacy and safety outcomes for ssCAR-Ts

Country	Study ID	*n*	Median ae	Male, *n* (%)	High-risk cytogenetics, *n* (%)	Prior therapy	ssCAR-Ts dose	ssCAR-Ts frequency	Study design	Endpoints reported	Sponsor	NCT number
**China**	Kang 2019 [38]	7	33 (20–59)	8 (61.5)	NR	3 (IQR, 1, range, 2–5)	Patients received 5 × 10^6^ cells/kg	Infused over three consecutive days (doses were split as 10%, 30%, and 60%)	NR	CR, CRS, CRES, IL-6 (peak and AUC_0_–*T*_max_), CAR-T expansion	NR	NR
**China**	Chen 2020 [27]	3	36 (36–48)	1 (33)	Ph+ 2/3 (66.7)	3 [3,4] (range, 1– 4)	Patients received 5 × 10^6^ cells/kg of ssCAR-T. Patient 2 received an additional 1 × 10^7^ cells/kg intrathecally	Infused over three consecutive days	Phase 1, open-label, case series	CR, OR, IrAE (CRS, ICANS), MRD, infections, GVHD, CNS response, CAR-T cell expansion and persistence, cytokine levels (e.g., IL-6), adverse events (e.g., neutropenia).	National Natural Science NCRCH Jiangsu Province Gusu Key Medical Talent Program	NCT03064269
**China**	Gong 2022 [26]	61	32 (9–73, IQR, 19.50–45.50)	30 (49.18)	27 (44.3)#	3 (IQR, 2,4, range, 1–10)	5 × 10^6^ cells/kg	Infused over two or three days (either 10%/30%/60% or 40%/60% splits)	Phase 2, single-arm clinical trial	OR, CR, PR, CRS, mCRS, sCRS, DOR, OS, ICANS, MRD, BMDB.		NCT03275493
**China**	Mao 2021 [36]	12	NR (43.41 ± 8.83)*	7(58.3)	NR	NR	5 × 10^6^ cells/kg	Infused over three consecutive days	Prospective observational, Phase 1 clinical study	IrAE (CRS, ICANS), OR, CR, PR, hyperthermia, inflammatory factors, muscle pain, headache, nausea, vomit, diarrhea, neurological impairment.	NR	NCT03064269
**China**	Li 2022 [39]	78	Low TB 32 (range 10–73) High TB 30 (range 9–68)	38 (48.72)	24 (30.8)$: Ph+ 14 (17.9), TP53 3 (3.8)	≥3 (70.5%)	5 × 10^6^ cells/kg	Infused over three split doses of 10%, 30%, and 60%	Phase 1/2, multicenter, open-label clinical trial	CR, MRD-negative CR, OS, EFS, CRS, neurotoxicity, cytokine levels (IL-6, IL-2, TNF-α, IFN-γ), CAR-T expansion	Chinese national and Jiangsu provincial research grants	NCT03919240
**China**	Kang 2023 [25]	47	33 (9–64)	24 (51.06)	Ph+ 12 (25.5); TP53 1 (2.1)	3 (range, 2–8)	5 × 10^6^ cells/kg	NR	Phase 1/2, multicenter, open-label clinical trial	OR, CR, PR, PFS, CRS, temp change, IFs, AEs (neutropenia, thrombocytopenia, CRS, ICANS), and complications (hyperthermia, increase in inflammatory factors, muscle pain, headache, nausea, neurological impairment).	The First Affiliated Hospital of Soochow University	NCT03919240
**China**	Xue 2025 [37]	17	39 years (IQR, 20–51)	9 (53)	8 (47.1) $: Ph+ 3 (17.6)	2 (IQR, 1–2.5)	Low-dose: 1 × 10^6^ cells/kg, medium-dose: 5 × 10^6^ cells/kg, high-dose: 10 × 10^6^ cells/kg, expansion phase: 1 × 10^6^ cells/kg	Infused over 3 split doses of 10%, 30%, and 60% at 1 × 10^6^, once daily for 3 days	Phase 1, single-arm, dose-escalation and expansion clinical trial	OR, MRD negativity, DOR, PFS, AEs (lymphocytopenia, neutropenia, leukopenia, anemia, thrombocytopenia), CRS, and ICANS.	Shanghai Unicar-Therapy Bio-Medicine Technology Co., Ltd.	NCT04825496

Abbreviations: AEs, adverse events; AUC_0_–*T*_max_, area under the curve from time zero to maximum concentration; BMDB, bone marrow disease burden; CAR-T, chimeric antigen receptor T-cell; CNS, central nervous system; CR, complete response; CRS, cytokine release syndrome; CRES, cytokine release syndrome-related events, DOR, duration of response; EFS, event free survival; GVHD, graft-versus-host disease; IFs, inflammatory factors; IL-6, interleukin-6; IrAE, immune-related adverse event; mCRS, mild cytokine release syndrome; MRD, minimal residual disease; NR, not reported; OS, overall survival; OR, overall response; Ph+, Philadelphia chromosome-positive; PR, partial response; PFS, progression-free survival; sCRS, severe cytokine release syndrome; TB, tumor burden; TP53, tumor protein p53 gene mutation. *Asterisk denotes mean age and standard deviation, as median age and range were not available. #Hash denotes high-risk (poor) cytogenetics defined per NCCN Guidelines v1.2021, including Ph+/BCR-ABL1, KMT2A rearrangement, hypodiploidy, complex karyotype, BCR-ABL1-like ALL, iAMP21, TCF3-HLF, and IKZF1 alterations. $Dollar indicates poor cytogenetics, including Ph+/BCR-ABL1 and TP53 mutation (Li and Xue) and BCR-ABL1-like ALL (Xue only).

ssCAR-T therapies were shown to retain the favorable efficacy of cCAR-Ts, as reported by studies such as Mao and Xu [36], Gong et al. [26], and Chen et al. [27], demonstrating an early complete response rate (CR) of 83%, 85%, and 100%, respectively, within a month after therapy. On the other hand, Xue et al. [37] reported lower CR rates of 44% and 43% for low and intermediate dose groups, respectively, compromising the pooled estimate of 84% with 95% CI ranging from 78% to 89% (Figure 2A). Furthermore, Kang et al. [38] reported a CR of 100% within all treated patients; however, this result was excluded from the analysis due to a non-specified duration of follow-up. Additionally, the prediction interval for the pooled CR rate ranged from 13% to 98%, indicating a high degree of variability in the effect sizes.

Assessment of residual disease across included studies was limited to MRD negative CR, which focuses on residual blast populations without considering immunophenotypes, nor identifying quiescent leukemic stem cells (qLSCs). These qLSCs may persist below detection limit, residing in protective bone marrow niches and serve as reservoir driving B-ALL relapse as supported by observed patterns of relapse. In other words, confirmation of MRD negativity by flow cytometry does not necessarily guarantee the elimination of LSCs. Chen et al. reported relapse as early as 3 months in a patient who initially achieved CSF clearance, which was attributed to the central nervous system acting as a sanctuary site with limited CAR-T cell penetrability, suggesting the persistence of LSCs in immunologically privileged niches. Moreover, Xue et al. reported a patient experienced MRD-positive relapse at 12 months despite initial MRD negativity. This relapse pattern is likely caused by LSCs that evade ssCAR-T-19-mediated killing via down-regulating CD19 antigens, entering a dormant condition, and immune shielding from the microenvironment. Future studies incorporating LSC-directed assays, next-generation sequencing-based MRD detection, or single-cell profiling will be necessary to evaluate whether ssCAR-Ts are successful in eliminating qLSCs.

Furthermore, the reported DOR data further reinforce the long-term efficacy of ssCAR-T, with a median DOR of 25.8 months, aligning findings by Gong et al. [26], who showed 56.26% of patients maintaining CR following 36 months of therapy. While PFS and OS data are still emerging, the studies show promising signs of efficacy in extending survival. Despite Kang et al. [25] observing no significant differences in median PFS, ssCAR-Ts demonstrated a significantly higher 3-month PFS (82.3%) compared with cCAR-Ts (66.9%), indicating that the ssCAR-Ts group may be more likely to experience therapeutic benefit sooner. According to Anagnostou et al. [40], anti-CD19 CAR-T therapy resulted in a pooled CR rate of 80% (95% CI: 75.5%–84.8%) after one year. In comparison, our analysis, which evaluates response at 1 month, offers promising OR and CR as early indicators of efficacy, potentially reflecting a quicker onset of therapeutic benefit that could foreshadow similar long-term outcomes.

ssCAR-Ts appear to have a promising efficacy profile, but relapse remains a concern in patients with low CAR-T cell expansion or extramedullary disease; thus, additional studies with longer follow-ups and a more detailed safety profile are necessary to confirm these findings. The high OR rates, DOR, and improved safety profile of ssCAR-Ts suggest that it has the potential to preserve the essence of cCAR-Ts’ efficacy in the treatment of r/r B-ALL. Nevertheless, the limitations of these studies and the absence of comprehensive survival data underscore the need for further research into ssCAR-Ts’ long-term efficacy and safety.

**Figure 3:**
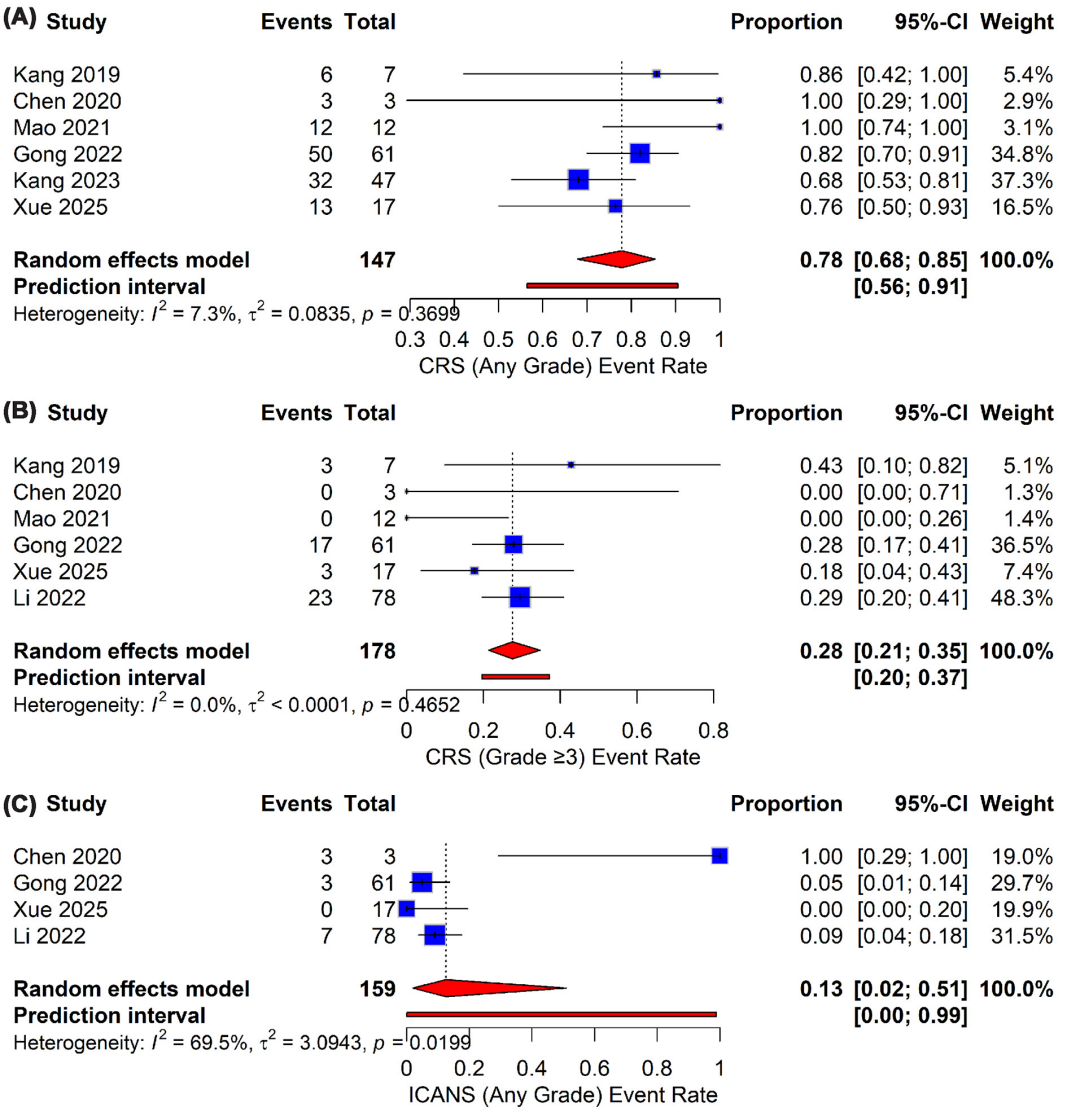
Pooled incidence of immune-related adverse events following ssCAR-T-19 therapy. Forest plots showing the proportion of patients developing (**A**) CRS of any grade, (**B**) severe CRS (grade ≥3), and (**C**) ICANS of any grade.

In terms of safety, ssCAR-Ts have demonstrated a notable incidence of CRS, but these instances are generally manageable under most circumstances. The outcomes for CRS any-grade were reported in all of the included studies, with rates below 80% observed by Kang et al. [25] and Xue et al. [37], whereas Gong et al. [26] reported 82%, yielding a pooled event rate of 78% (95% CI: 68%–85%, Figure 3A). Nevertheless, the incidence of severe CRS (grade 3 or higher) among leukemic patients administered ssCAR-Ts was remarkably low, with rates ranging from 0% to 43%. This led to a pooled figure of just 28% (95% CI: 21%–35%; Figure 3B). The prediction interval ranged from 56% to 91% for CRS (any grade), indicating moderate consistency; however, the narrower interval (20%–37%) for severe grade CRS suggests enhanced certainty, highlighting the contrasting reliability of these results. These outcomes should be interpreted within the context of this study design. On the contrary, ICANS events were rare for ssCAR-T therapy. Xue et al. [37], Mao and Xu [36], and Kang et al. [25] reported any-grade ICANS rates in the range of 0% to 8%, aligning with an impressive, pooled rate of 13% (95% CI: 2%–51%; Figure 3C). Notably, severe ICANS (grade ≥3) was not detected across all included studies.

When these safety results are put together with previously published large-scale evidence, the differences become noticeable. In the meta-analysis by Anagnostou et al., the pooled rate of any-grade CRS across 35 studies of anti-CD19 CAR-T therapy was reported as 82% (95% CI: 71.6%–90.7%), with grade 3 or higher CRS occurring in 26% (95% CI: 18.5%–34.1%) [40]. ssCAR-Ts in the present analysis exhibited a comparable incidence of any-grade CRS rates and grade 3 or higher CRS (Figure 3B). On the other hand, for neurotoxicity, Anagnostou et al. reported any-grade events in 29% and grade 3 or higher neurotoxicity in 12% of patients, whereas ssCAR-Ts cohorts in our meta-analysis demonstrated markedly better neurotoxicity profiles (Figure 3C) and complete absence of grade 3 or higher events.

Since all included studies were single-arm, non-randomized trials, the initial overall certainty of evidence was considered low for all pooled outcomes, in line with GRADE guidelines [33]. With regard to the five downgrading domains, no serious concerns were raised about CR, OR, and CRS at any grade, and CRS grade ≥3 (Table 2). This resulted from the following factors: absence or negligible statistical heterogeneity (*I*^2^ = 0.0%–7.3%), all studies directly assessed the target population (r/r B-ALL patients receiving ssCAR-T-19) without major concern for indirectness, and pooled estimates demonstrated good precision with adequately narrow confidence intervals. Although CRS grading criteria varied across studies, this was considered as methodological nuances rather than real indirectness. Funnel plots for CR, CRS (any grade), and CRS (grade ≥3) revealed all studies falling within the expected funnel boundaries, suggesting no evidence of publication bias (Supplementary Figure 1a–c). Nevertheless, the limited number of involved studies per outcome (*n* ≤6) undermines the reliability of these interpretations and constrains judgement over asymmetry. For other outcomes, the small number of studies (*n* <5) per outcome prevented a formal assessment of publication bias; therefore, outcomes cannot be downgraded based on formal bias test. Yet, all included studies had pre-specified outcome measures, minimizing selective reporting risk. Therefore, publication bias was classified as undetected rather than absent, and the evidence was not downgraded for this domain. Since no downgrading was applied to these four outcomes, and upgrading domains were also inapplicable due to the single-arm, non-comparative design, hence the final overall of certainty of evidence remained as low.

**Table 2:** GRADE assessment of the certainty of evidence for the included studies

Outcome domain	Number of participants	No. of studies	Phase of investigation	Initial rating	Downgrading domains					Upgrading domains			Final certainty in evidence
					Study limitations	Inconsis- tency	Indirec- tness	Impreci- sion	Public- ation bias	Effect size	Exposure- response gradient	Residual confounding	
**OR at 1 month**	136	4	Phase 1/2	Low	✓	✓	✓	✓	✓	✘	✘	✘	Low
**CR at 1 month**	171	5	Phase 1	Low	✓	✓	✓	✓	✓	✘	✘	✘	Low
**CRS (any grade)**	147	6	Phase 1	Low	✓	✓	✓	✓	✓	✘	✘	✘	Low
**CRS (grade ≥3)**	178	6	Phase 1	Low	✓	✓	✓	✓	✓	✘	✘	✘	Low
**ICANS (any grade)**	159	4	Phase 1	Low	✓	✘	✓	✘	✓	✘	✘	✘	Very low
**ICANS (grade ≥3)**	67	3	Phase 1	Low	✓	✓	✓	✓	✓	✘	✘	✘	Low

A tick (✓) indicates that the criteria have been met or satisfied, including meeting the expected consistency, directness, precision, and absence of significant limitations or bias, whereas a ballot x (X) specifies these criteria were not met.

**Figure 4:**
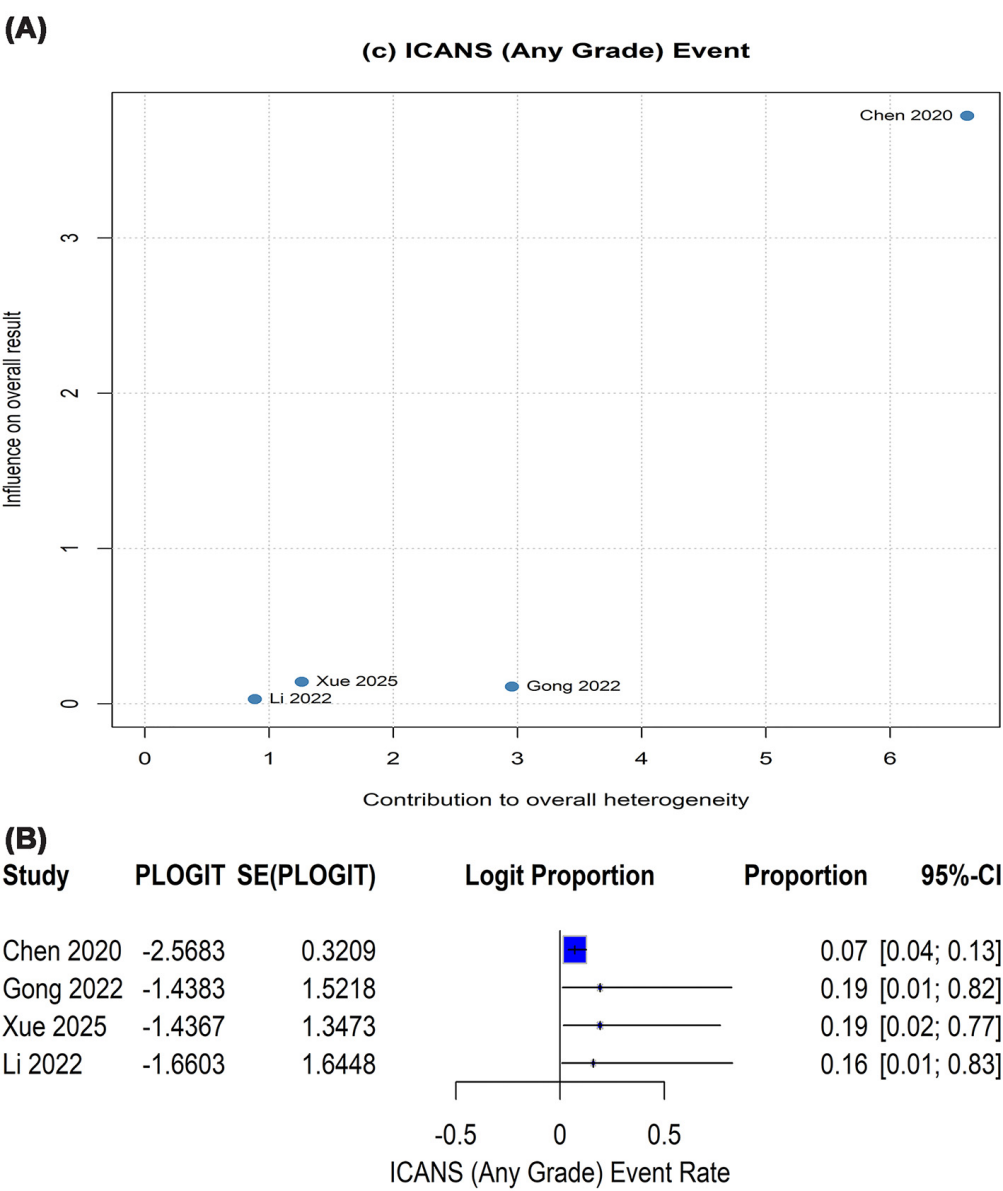
Sensitivity analyses exploring sources of heterogeneity in pooled ICANS estimates. (**A**) Baujat plot identifying studies contributing disproportionately to overall heterogeneity and influence on the pooled effect. (**B**) Leave-one-out analysis showing the impact of sequential study omission on the pooled estimate.

Additionally, for ICANS (any grade), two domains deserved downgrading, resulting in a very low certainty of evidence (Table 2). First, significant heterogeneity (*I*^2^ = 69.5%, *P* = 0.0199) was remarked when pooling ICANS (any grade), warranting further investigation through sensitivity analysis to identify its source and impact on the pooled estimate. The Baujat plot identified Chen 2020 as the primary source of heterogeneity, as pointed at the upper-right quadrant (Figure 4A), contributing disproportionately to both the overall heterogeneity and influence on the pooled result. Leave-one-out iterations that retained Chen’s study while excluding other studies converged on higher and less consistent ICANS proportions (0.16–0.19; 95% CI: 0.01–0.83) (Figure 4B). Leave-one-out sensitivity analysis confirmed that omitting Chen’s study resolved the observed heterogeneity and resulted in lower, stable, and robust pooled estimate (0.07; 95% CI: 0.04–0.13) (Figure 4B). Second, imprecision was a concern as the pooled estimate had a broad confidence interval for ICANS (0.02–0.51) with prediction interval covering nearly the entire probability range (0.00–0.99), reflecting high uncertainty in the true ICANS rate even with individually precise study estimates. Similar to other outcomes, no further upgrading beyond post-downgrading, so the final certainty ratings remained unaffected.

There was some variation in CRS and ICANS grading criteria among the included studies (Supplementary Table S2). ASTCT 2019 consensus grading criteria were applied to both CRS and ICANS in two studies by Chen et al. and Xue et al. Gong et al. followed a dual approach combining ASTCT 2019 criteria with local institutional criteria that comprised NCCN and CTCAE guidelines. On the other hand, Mao and Xu followed alternative CRS grading based on the National Cancer Institute’s Common Terminology Criteria for Adverse Events (CTCAE), whereas ICANS grading was not reported. Neither Kang et al. 2019 nor Kang et al. 2023’s conference reports provided details on the grading system. Overall, half of the included studies applied ASTCT criteria, the current CAR-T-specific standard for CRS and ICANS grading, whereas one of these studies relied completely on CTCAE frameworks, which are not directly comparable and introduce some grading heterogeneity. It is therefore pertinent to consider this variability in grading definitions and thresholds when interpreting CRS and ICANS incidence rates across studies.

Together, these findings suggest that ssCAR-Ts may offer meaningful improvements in the safety profile, especially in terms of severe CRS and neurotoxicity, when compared with earlier generations of anti-CD19 CAR-T constructs. In the event that this improved tolerability is successfully validated in larger and longer-term studies, CAR-T therapy could be shifted from a risk-benefit analysis toward a broader and safer application in the clinic.

The peak cytokine levels observed in patients post-ssCAR-T-19 infusion, particularly IL-6, IL-10, and IFN-γ, positively correlate with CRS severity. Gong et al. [26] reported increased inflammatory markers in patients with severe CRS compared with mild CRS, with IL-10 being about 12-fold increased (*P* <0.001), and IFN-γ was nearly 17-fold elevated in severe cases (*P* <0.0001). Meanwhile, CRP levels were more than twofold higher in severe CRS patients (*P* <0.01 for both). These results were in alignment with previous significant findings by Kang et al. [38], which supports that ssCAR-Ts therapy may reduce the inflammatory burden compared with cCAR-Ts. On the other hand, Xue et al. [37] did not consistently correlate peak cytokine levels with CRS severity, suggesting that other factors may be responsible.

The dynamics of inflammatory markers such as IL-6, CRP, and ferritin in the post-infusion period underscore the acute-phase inflammatory response to ssCAR-T-19 therapy. Following infusion, cytokines and markers such as IL-6, ferritin, and CRP peak within 3–6 days and decline over a ten-day period, as reported by Mao and Xu [36]. Further evidence was provided by Xue et al. [37], where IL-6 peaks in the low-dose group with a median of 7 days (IQR 7–9), and ferritin and CRP exhibit similar patterns.

In addition, Gong et al. [26] observed that severe CRS was significantly more likely to begin earlier than mild cases, with patients experiencing the onset of severe symptoms on average of one day (IQR: 1–2) versus two days for mild symptoms (IQR: 1–4) (*P* = 0.002). Both groups reached their peak CRS effects on day 4, while the median remission time for both mCRS and sCRS was comparable (day 8). In summary, ssCAR-Ts in the present analysis deliver efficacy results that closely mirror those of the landmark previous meta-analysis of cCAR-Ts, while adding a layer of safety to the traditional platform, highlighting a clear therapeutic potential of this next-generation engineering strategy.

Aside from CRS and ICANS, CAR-T cell therapy can result in a broader spectrum of complications including organ dysfunction, prolonged cytopenias, and infectious complications secondary to immunosuppression [41–43]. Among the included studies, hematological toxicities were reported (grade 3–4 neutropenia 29.79%–94%, thrombocytopenia 36%–88%), hepatotoxicity (elevated AST/ALT by up to 65%), coagulopathy, electrolyte disturbances, and infections. Xue et al. also reported universal B-cell aplasia and hypogammaglobulinemia (47%), implying on-target, off-tumor effects of CD19-targeting CAR-Ts. Cytopenia was also observed as a serious complication that caused two deaths from septic shock secondary to prolonged neutropenia. Nonetheless, most of these toxicities were strongly correlated with CRS severity (Gong et al.), indicating that they are downstream consequences of the cytokine-mediated inflammatory response rather than independently caused by CAR-Ts. A shortcoming of the current evidence is that it exclusively focuses on CRS and ICANS, both of which were reported consistently; thus, future trials should present standardized reporting on other relevant adverse events to facilitate robust analysis of ssCAR-Ts impact on improving safety profile over cCAR-Ts.

**Figure 5:**
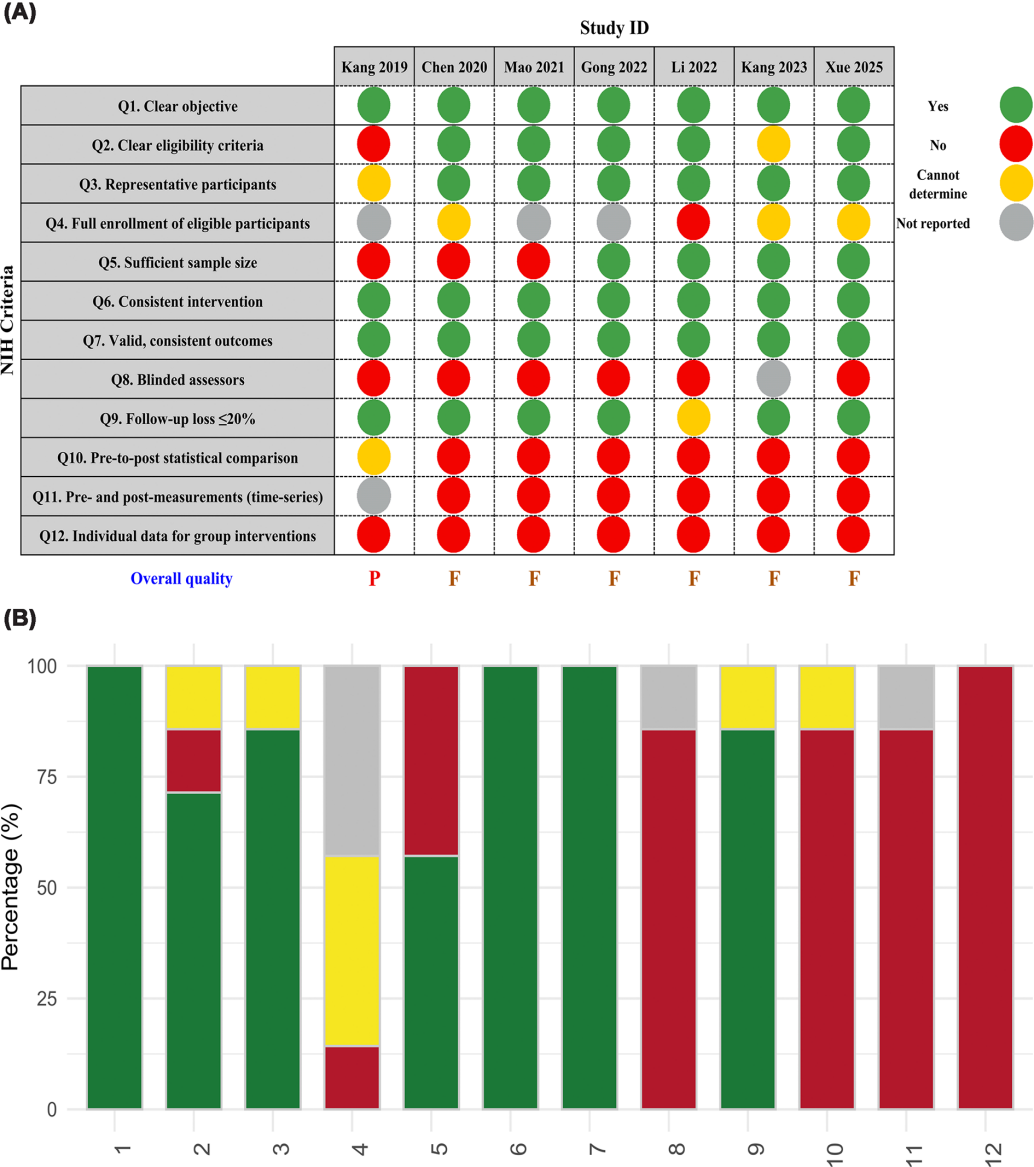
Methodological quality of included studies assessed using the NIH quality assessment tool for pre–post studies with no control group. (**A**) Study-level quality ratings across 12 NIH criteria. (**B**) Overall distribution of risk-of-bias judgements across studies.

After the subjective evaluation of included studies in accordance with the NIH criteria, it appears that they adhered to different key methodologies and received various quality ratings (Figure 5A). This rating showed that the majority of studies exhibited fair adherence to essential aspects of the study design; however, gaps in areas such as statistical analysis and participant selection raised some concerns about potential underlying bias. However, since studies were single arm, inherent lack in blinding may have introduced participant and observer biases, potentially impacting the overall validity and generalizability of the findings. On the other hand, Kang et al. [38] achieved poor ratings, highlighting methodological concerns as they involved insufficient statistical analysis, which contributed to considerable bias and undermined the study validity. Figure 5B highlights that there were five shared weaknesses in almost all studies, including the full enrolment of eligible participants, blinding, along with the lack of pre-to-post statistical comparisons, repeated measurements of outcomes, individual data. These flaws have already impacted the conclusions, calling for further iterations that address such weaknesses to improve their internal validity and to ensure the production of high-quality, generalizable evidence.

In interpreting the findings of the present study, it is important to acknowledge that there are important limitations. All data were collected solely from China, limiting the external validity of the findings in view of disparities in healthcare systems, environmental, and socioeconomic aspects, not to mention clinical practices. Since no ethnic diversity was present in the present study, the outcomes may not be generalizable to more diverse and multiethnic populations. Moreover, pooling estimates from seven studies, which included 178 patients, is a primary limit on statistical power in our analysis and can result in unreliable or inaccurate estimates of effect size. Hence, it is challenging to generalize these estimates, as they are prone to outliers and restricted by demographic heterogeneity.

A further concern is that some included studies were undertaken in collaboration with funding bodies, which may have introduced potential sponsorship bias despite disclosures. As such, the current conclusions require further validation by an independent, large-scale, multicenter study across diverse populations. Moreover, as discussed earlier, heterogeneity was noticed in grading criteria applied to rank CRS and ICANS severity across studies, presenting a source of clinical inconsistency and urging future studies to adopt standardized assessment criteria to improve cross-study comparability. We also acknowledge GRADE assessment as a judgment-based approach and have maximized transparency by presenting forest plots, raw data, and direction tallies alongside our grading decisions in Table 2. It is therefore prudent to view current findings as preliminary, and rather than drawing any definitive conclusions, it is imperative to rely on larger prospective studies and randomized controlled trials.

The current analysis is also limited by lack of comprehensive cytogenetic subtyping across included studies. Although high-risk cytogenetic data were reported in five out of seven studies, the reporting varied greatly, with some studies providing overall high-risk proportions and others describing individual abnormalities, including Philadelphia chromosome positivity (ranging between 17.6% and 25.5% in studies with >10 patients) and TP53 mutations. Neither of the included studies reported efficacy or safety outcomes stratified by cytogenetic subtypes, limiting the feasibility of conducting subgroup analyses. There are, however, prior evidence showing that anti-CD19 CAR-Ts achieved comparable remission rates and survival outcomes across cytogenetic risk groups, including high-risk subtypes, including Philadelphia chromosome-positive (Ph^+^) and lysine methyltransferase 2A (KMT2A)-rearranged ALL [44]. Confirming how far these outcomes extend to ssCAR-Ts therapy calls for further prospective trials comprising pre-specified cytogenetic subgroup analyses.

## Conclusion

ssCAR-Ts look promising, particularly in terms of overall remission rates and mitigating neurological toxicities; however, further follow-up is necessary to demonstrate whether they offer superior long-term survival benefits. Although the certainty of evidence was mostly low for outcomes associated with ssCAR-Ts' safety and efficacy, ssCAR-Ts may be a more attractive option in clinical practice for their potentially lower ICANS event rates, particularly when considering patients with pre-existing neurological conditions for whom cCAR-T therapy is contraindicated.

## Supplementary Material

Supplementary Figure S1 and Tables S1-S2

## Data Availability

All data analyzed in this study were extracted from the included primary studies and are presented within the manuscript, primarily in the forest plots (Figures 2 and 3) and Table 1. Further details are available from the corresponding author upon reasonable request.

## Open Access

Open access for this article was enabled by the participation of Griffith University in an all-inclusive Read & Publish agreement with Portland Press and the Biochemical Society under an agreement with CAUL.

## Abbreviations

ASTCT: American Society for Transplantation and Cellular Therapy
BBB: blood-brain barrier
cCAR-T: conventional CAR-T
CAR: chimeric antigen receptor
CR: complete response
CRP: C-reactive protein
CRS: cytokine release syndrome
CSF: cerebrospinal fluid
CTCAE: Common Terminology Criteria for Adverse Events
DAMPs: damage-associated molecular patterns
DOR: duration of response
Emtree: Embase subject headings
GRADE: grading of recommendations, assessment, development and evaluations
ICANS: immune effector cell-associated neurotoxicity syndrome
IrAEs: immune-related adverse events
IFN-γ: interferon-γ
IL-6: interleukin-6
IQR: interquartile range
MRD: minimal residual disease
NCCN: National Comprehensive Cancer Network
NCI: National Cancer Institute
NIH: National Institutes of Health
OR: overall response
OR: overall response
PFS: progression-free survival
PICO: population, intervention, comparator, outcome
r/r B-ALL: refractory B-cell acute lymphoblastic leukemia
shRNA: short hairpin RNA
ssCAR-T: shRNA-engineered CAR-T cells
ssCAR-T-19: shRNA-engineered anti-CD19 CAR-T cells
qLSCs: quiescent leukemic stem cells
