# Supplementary material for: A systematic review and meta-analysis of shRNA–IL-6-engineered CAR-T cells for B-cell acute lymphoblastic leukemia: a stepping stone toward risk-free immunotherapy
Source: Biosci Rep. 2026 May 22;46(6):BSR20260143. doi: 10.1042/BSR20260143 (PMC13199798; doi:10.1042/BSR20260143)
Supplement: Supplementary Figure S1 and Tables S1-S2 [file BSR-2026-0143_supp.pdf]

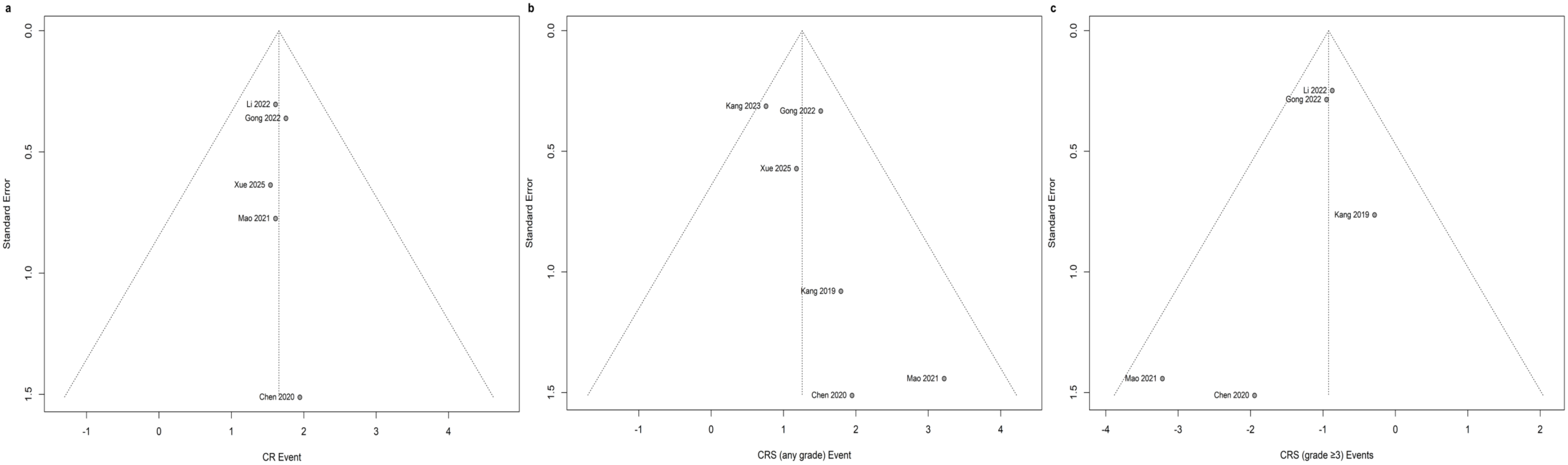

Supp. Fig. 1: (a) Funnel plot assessing publication bias for CR, (b) CRS (any grade), and (c) CRS (grade ≥ 3) rates. Effect sizes are presented as logit-transformed proportions. The vertical dashed line represents the pooled logit estimate; dotted lines represent the 95% pseudo-confidence interval boundaries.

Table 1. Database-specific search strategies and syntax used for systematic literature retrieval

Database	Database-specific search syntax
**PubMed**	(("shRNA"[tiab] OR "short hairpin RNA"[tiab] OR "RNA interference"[tiab] OR "RNAi"[tiab] OR "gene silencing"[tiab]) OR "RNA Interference"[MeSH Terms]) AND (("CAR T cell"[tiab] OR "CAR-T"[tiab] OR "chimeric antigen receptor T cell"[tiab]) OR "Receptors, Chimeric Antigen"[MeSH Terms]) AND (("leukemia"[tiab] OR "lymphoma"[tiab] OR "myeloma"[tiab] OR "hematologic malignancy"[tiab]) OR "Hematologic Neoplasms"[MeSH Terms])
**Embase**	('shRNA'/exp OR 'short hairpin RNA':ab,ti,kw OR 'RNA interference'/exp OR 'RNA interference':ab,ti,kw OR 'RNAi':ab,ti,kw OR 'gene silencing':ab,ti,kw) AND ('chimeric antigen receptor'/exp OR 'CAR T cell':ab,ti,kw OR 'CAR-T':ab,ti,kw OR 'chimeric antigen receptor T cell':ab,ti,kw) AND ('hematologic malignancy'/exp OR 'leukemia':ab,ti,kw OR 'lymphoma':ab,ti,kw OR 'myeloma':ab,ti,kw)
**Scopus**	TITLE-ABS-KEY ("shRNA" OR "short hairpin RNA" OR "RNA interference" OR "RNAi" OR "gene silencing") AND TITLE-ABS-KEY ("CAR T cell" OR "CAR-T" OR "chimeric antigen receptor T cell") AND TITLE-ABS-KEY ("leukemia" OR "lymphoma" OR "myeloma" OR "hematologic malignancy")
**Web of Science**	(TI=("shRNA" OR "short hairpin RNA" OR "RNA interference" OR "RNAi" OR "gene silencing") AND TI=("CAR T cell" OR "CAR-T" OR "chimeric antigen receptor T cell") AND TI=("leukemia" OR "lymphoma" OR "myeloma" OR "hematologic malignancy")) OR (AB=("shRNA" OR "short hairpin RNA" OR "RNA interference" OR "RNAi" OR "gene silencing") AND AB=("CAR T cell" OR "CAR-T" OR "chimeric antigen receptor T cell") AND AB=("leukemia" OR "lymphoma" OR "myeloma" OR "hematologic malignancy"))

Table 2: Grading Criteria Applied for CRS and ICANS Across Included Studies

Study	CRS Grading System	ICANS Grading System
**Kang et al. 2019**	Not specified	NA
**Chen et al. 2020**	ASTCT 2019	
**Mao & Xu 2021**	NCI-CTCAE	NA
**Li et al. 2022**	CTCAE version 4.0.3	NA
**Gong et al. 2022**	ASTCT 2019 + local institutional criteria*	NA
**Kang et al. 2023**	CTCAE version 4.0.3	NA
**Xue et al. 2025**	ASTCT 2019	

ASTCT: American Society for Transplantation and Cellular Therapy. NCI-CTCAE: National Cancer Institute Common Terminology Criteria for Adverse Events. *This local criterion was modified from the National Comprehensive Cancer Network (NCCN) Guidelines (version 1.2021) and CTCAE version 5.0. NA: This study has not conducted ICANS grading
